# The Accuracy of Wrist Skin Temperature in Detecting Ovulation Compared to Basal Body Temperature: Prospective Comparative Diagnostic Accuracy Study

**DOI:** 10.2196/20710

**Published:** 2021-06-08

**Authors:** Tracy Y Zhu, Martina Rothenbühler, Györgyi Hamvas, Anja Hofmann, JoEllen Welter, Maike Kahr, Nina Kimmich, Mohaned Shilaih, Brigitte Leeners

**Affiliations:** 1 Department of Reproductive Endocrinology University Hospital Zurich Zurich Switzerland; 2 Ava AG Zurich Switzerland; 3 Department of Obstetrics University Hospital Zurich Zurich Switzerland; 4 University of Zurich Zurich Switzerland

**Keywords:** ovulation, basal body temperature, BBT, oral temperature, wrist skin temperature, diagnostic accuracy, thermometer, fertility, menstruation, wearable, sensor, mobile phone

## Abstract

**Background:**

As a daily point measurement, basal body temperature (BBT) might not be able to capture the temperature shift in the menstrual cycle because a single temperature measurement is present on the sliding scale of the circadian rhythm. Wrist skin temperature measured continuously during sleep has the potential to overcome this limitation.

**Objective:**

This study compares the diagnostic accuracy of these two temperatures for detecting ovulation and to investigate the correlation and agreement between these two temperatures in describing thermal changes in menstrual cycles.

**Methods:**

This prospective study included 193 cycles (170 ovulatory and 23 anovulatory) collected from 57 healthy women. Participants wore a wearable device (Ava Fertility Tracker bracelet 2.0) that continuously measured the wrist skin temperature during sleep. Daily BBT was measured orally and immediately upon waking up using a computerized fertility tracker with a digital thermometer (Lady-Comp). An at-home luteinizing hormone test was used as the reference standard for ovulation. The diagnostic accuracy of using at least one temperature shift detected by the two temperatures in detecting ovulation was evaluated. For ovulatory cycles, repeated measures correlation was used to examine the correlation between the two temperatures, and mixed effect models were used to determine the agreement between the two temperature curves at different menstrual phases.

**Results:**

Wrist skin temperature was more sensitive than BBT (sensitivity 0.62 vs 0.23; *P*<.001) and had a higher true-positive rate (54.9% vs 20.2%) for detecting ovulation; however, it also had a higher false-positive rate (8.8% vs 3.6%), resulting in lower specificity (0.26 vs 0.70; *P*=.002). The probability that ovulation occurred when at least one temperature shift was detected was 86.2% for wrist skin temperature and 84.8% for BBT. Both temperatures had low negative predictive values (8.8% for wrist skin temperature and 10.9% for BBT). Significant positive correlation between the two temperatures was only found in the follicular phase (*rmcorr* correlation coefficient=0.294; *P*=.001). Both temperatures increased during the postovulatory phase with a greater increase in the wrist skin temperature (range of increase: 0.50 °C vs 0.20 °C). During the menstrual phase, the wrist skin temperature exhibited a greater and more rapid decrease (from 36.13 °C to 35.80 °C) than BBT (from 36.31 °C to 36.27 °C). During the preovulatory phase, there were minimal changes in both temperatures and small variations in the estimated daily difference between the two temperatures, indicating an agreement between the two curves.

**Conclusions:**

For women interested in maximizing the chances of pregnancy, wrist skin temperature continuously measured during sleep is more sensitive than BBT for detecting ovulation. The difference in the diagnostic accuracy of these methods was likely attributed to the greater temperature increase in the postovulatory phase and greater temperature decrease during the menstrual phase for the wrist skin temperatures.

## Introduction

### Background

Basal body temperature (BBT) is the lowest body temperature in the circadian rhythm. Monitoring BBT is one of the simplest and least invasive methods for determining the occurrence of ovulation and estimating its timing during the menstrual cycle [1]. In most women, BBT reaches its lowest point in each cycle just before ovulation and increases in the luteal phase because of the thermogenic properties of progesterone [2]. This physiological change is known as a temperature shift that presents as a biphasic pattern on the BBT curve [3].

Oral temperature taken immediately upon waking is widely used for measuring BBT by women who are interested in tracking their menstrual cycles or women who are trying to conceive because it is easy to use and noninvasive [4]. As a daily point measurement, BBT curves are sensitive to missing values and the time of measurement; a temperature shift may go undetected because a rise in body temperature may not have occurred yet at the time of the measurement [5]. In addition, lifestyle factors may strongly influence the reliability of this method [6]. A number of devices offering continuous temperature measurements at different body sites have been developed over the past decade [7-13]. In our previous studies, we have shown that continuously measured wrist skin temperature during sleep also presented a biphasic pattern in menstrual cycles, with 82% of the observed cycles having a sustained 3-day temperature shift [9,12].

### Objectives

The primary objective of this study is to determine whether continuously measured wrist skin temperature during sleep was more accurate in detecting ovulation than BBT measured by oral temperature, using luteinizing hormone (LH) tests as the standard reference. The secondary objective is to investigate the correlation and agreement between these two temperatures in describing thermal changes in menstrual cycles.

## Methods

### Study Design and Participants

This prospective comparative diagnostic accuracy study was conducted from February to August 2019. The study was conducted in accordance with the Declaration of Helsinki and was approved by the Cantonal Ethics Committee of Zurich, Switzerland (BASEC-Nr 2016-02241). All participants provided written informed consent before any study procedures were performed.

A convenience sample of participants was recruited through social media advertisements and networks from January to February 2019. At the time of enrollment, the research staff assessed the eligibility of potential participants using a screening questionnaire. This assessment was conducted at the Department of Reproductive Endocrinology, University Hospital of Zurich. Healthy women who met the following criteria were considered eligible: aged 18-45 years, not currently on hormonal therapy, willing to comply with the study protocol for up to six cycles, not planning pregnancy within the following 6 months, and currently living in Switzerland. Women were excluded if they had problems wearing the bracelet, had difficulty understanding the study procedures, had any health-related issues potentially affecting their menstrual cycles, were taking any medication or other substances that could affect the menstrual cycles or any physiological parameters being studied, were working night shifts or frequently traveling between different time zones, had a sleeping disorder or slept less than 4 hours per night, or were actively breastfeeding. Eligibility criteria had no restrictions on the regularity or length of menstrual cycle.

Information on age, body weight, body height, race, and time since stopping hormonal contraception was collected after receiving informed consent. BMI was calculated as kg/m^2^. During the study period, participants measured continuous wrist skin temperature using the Ava Fertility Tracker, BBT using the Lady-Comp, and a home-based urine LH test using the ClearBlue Digital Ovulation Test (Swiss Precision Diagnostics GmbH). Participants received all the study materials, including detailed guidelines and study devices. The research staff provided participants with instructions on using the study devices and steps to be completed during the study. Contact details of the research staff and technical support staff for the Ava Fertility Tracker are provided. Participants were instructed to start all study measurements from the first day of enrollment, independent of their menstrual cycle day. This was done to enhance compliance and ensure that any issues were promptly resolved before the commencement of the next cycle.

### Continuous Wrist Skin Temperature and BBT Measurement

Participants wore the Ava Fertility Tracker bracelet (version 2.0, Ava AG) on the dorsal side of their wrist (always of the same arm) each night while sleeping. The bracelet measures four physiological parameters simultaneously: wrist skin temperature, heart rate, heart rate variability, and breathing rate. The latter three parameters were not of interest to this study and thus were not included. At least 4 hours of relatively uninterrupted sleep each night is required for the physiological parameters to stabilize according to the manufacturer’s packaging. The bracelet automatically saves physiological information every 10 seconds throughout the night. For this study, the first 90 minutes and the last 30 minutes of each night’s data were excluded to avoid disturbances of the falling asleep and waking up phases. Temperature data were smoothed using locally weighted scatterplot smoothing. The 99th percentile (stable maxima) was chosen out of several percentiles (10th, 50th, and 90th percentiles) as the daily wrist skin temperature in the final analyses [12]. During the initial interview, participants were shown how to synchronize the device with the complementary Ava app on their smartphones and were instructed to synchronize their data each morning after waking up. During synchronization, the anonymized device data were transferred to the server. After completion of the study, the research staff retrieved the wrist skin temperature data obtained during the study period from the server for the final analysis.

BBT was measured by Lady-Comp (Valley Electronics AG), which is a computerized fertility tracking device with a digital thermometer. The device includes a display panel that provides immediate temperature readings to its user. The participants measured their oral temperature using the device each morning immediately after waking up, before getting out of bed, and before starting any activity such as drinking water. BBT data were retrieved by connecting the device to a computer. For each participant, a file containing the BBT data obtained during the study period was downloaded from the manufacturer’s website. After completing the study, participants either retrieved the data and sent them to the research staff or they sent the device to the research staff, who retrieved the data. The device was returned to the participants after the data retrieval.

Participants recorded the first day of menstrual bleeding on both the Ava app and Lady-Comp. The first day of bleeding was defined as the first day of the cycle. In case of discrepancies, the date on the Ava app was used.

### LH Test

Participants performed a home-based urine LH test using the ClearBlue Digital Ovulation Test [14] for each cycle according to the manufacturer’s instructions. Home-based LH tests are widely used to detect ovulation and determine the fertile window [1,15]. For this study, the test was performed on prespecified days of the participant’s menstrual cycle. The starting day was calculated by identifying a participant’s average number of cycle days and then subtracting it by 17 days. After the initial test, the participant continued doing the LH test daily until a positive result, which was shown as *a stable smiling face* symbol on the device, or until the next menstruation. A positive result indicates an LH surge, which typically occurs 1 day before ovulation [13]. Participants reported the LH test results in the dedicated field of their Ava app. The day following the LH surge was defined as the day of ovulation. A cycle with a positive LH test was considered as an ovulatory cycle, and one with only negative LH tests was considered an anovulatory cycle. The LH test served as the reference standard for evaluating the diagnostic accuracy of the two temperatures.

### Statistical Analyses

This study was a subanalysis of a prospective diagnostic accuracy study that compared the 2 fertility tracking devices with the LH test. The hypothesis of the main study was that the Ava Fertility Tracker bracelet was equivalent to the Lady-Comp and LH tests in determining the ovulation day. The final analyses in the main study were restricted to ovulatory cycles. Assuming a clinically meaningful margin of ±2 days, SD of 3 days, a mean difference of 0, an intraclass correlation coefficient of 0.147, and three cycles per woman, 39 cycles from 13 women were required with 90% power and a one-sided α of .025. Assuming a 20% loss to follow-up and up to 50% of the cycles were excluded because of being anovulatory or missing data, a total of 58 women were planned for this study. This subanalysis study only used the temperatures collected by the 2 devices as index tests. All ovulatory and anovulatory cycles, except those affected by missing data, were included in this subanalysis study.

A simplified diagram presented the numbers of screened, eligible, consented, compliant, withdrawing, and lost to follow-up participants. Cycles with missing LH test results or ≥30% missing temperature measurements of either device were excluded from the final analysis. The baseline characteristics of the participants and their cycles were summarized using descriptive statistics. Continuous parameters were summarized as mean (SD), and categorical parameters were summarized as frequency (%). A temperature shift occurred if three temperature measurements were 0.2 °C above the highest value of the previous six measurements or of the previous five out of six measurements in case of missing values [1]. On the basis of this definition, multiple temperature shifts could be detected within a cycle. To avoid including variations in temperatures because of reasons other than the menstrual rhythm, such as sickness, we analyzed only those temperature shifts occurring during the last 14 days of a cycle. The total number of temperature shifts per cycle, the percentage of cycles showing at least one temperature shift, and the first day of the temperature shift relative to the day of ovulation were recorded. The diagnostic accuracy of using at least one temperature shift on the two temperature curves for detecting ovulation was evaluated using the LH test as the reference standard. Diagnostic accuracy measures included sensitivity, specificity, and predictive values, considering the nested design of the study.

The analyses of correlation and agreement between the two temperatures were performed only for ovulatory cycles because the ovulation day was required to separate the menstrual phases. Correlations between mean wrist skin temperature and mean BBT at the follicular and luteal phases were examined using repeated measures correlation (R package *rmcorr*). The *rmcorr* correlation coefficient (*r_rm_*) determines the common intraindividual association for paired measurements assessed on two or more occasions for multiple individuals [16]. Linear mixed effects models with random intercepts and random slopes were used to examine the agreement between the curves of the wrist skin temperatures and BBT. In these analyses, daily temperature measurements were nested within cycles, and the cycles were nested within the participants. Linear mixed effects models allow the modeling of repeated measurements, further accounting for correlated intraindividual and intracycle observations [12]. Daily temperature measurements were estimated from the models. The temperature curves at the cycle level were smoothed using locally weighted scatterplot smoothing. As curve patterns changed within a cycle, the agreement between curves was examined in three separate phases: (1) the menstrual phase referred to the period from day 1 to day 5 of a menstrual cycle; (2) the preovulatory phase referred to the period starting 10 days before ovulation and lasting through the day of ovulation; and (3) the postovulatory phase referred to the period starting 1 day after ovulation to 10 days after ovulation. A temperature curve was considered biphasic if at least one temperature shift was present. A curve was monophasic if a temperature shift was absent.

All statistical analyses were performed using the R software (version 3.6.0). All hypotheses were two-tailed. Statistical significance was set at *P*<.05.

## Results

### Participants and Cycles

**Figure 1:**
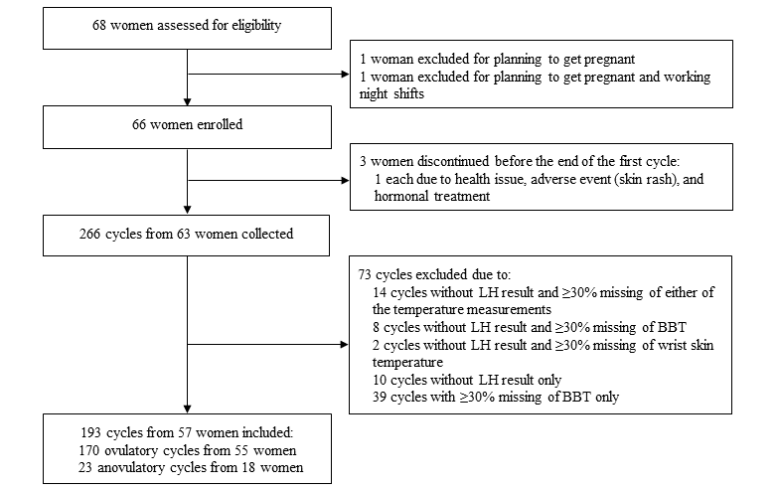
Study flowchart. BBT: basal body temperature; LH: luteinizing hormone.

In total, 266 cycles were collected from 63 women (Figure 1). The final analysis included 193 cycles of 57 women. A total of 88.1% (170/193) of cycles from 55 women were ovulatory (cycles with positive LH test), and 11.9% (23/193) from 18 women were anovulatory (cycles with only negative LH test). Table 1 presents the characteristics of the participants and their cycles. In total, 9% (2/23) anovulatory cycles were less than 24 days, and 13% (3/23) were more than 35 days. No ovulatory cycles were less than 24 days, but 4.1% (7/170) were more than 35 days. Most of the ovulatory cycles (146/170, 85.9%) had a luteal phase length between 11 days and 17 days and only 13.5% (23/170) were ≤10 days.

**Table 1:** Characteristics of the participants and their cycles.

Characteristics				Results
**Participant level (n=57)**				
	Age (years), mean (SD)			26.7 (4.2)
	Age (years), min-max			18-35
	**Age groups (years), n (%)**			
		18-20	5 (9)	
		21-25	19 (33)	
		26-30	23 (40)	
		31-35	10 (18)	
	Height (cm), mean (SD)			166.5 (6.0)
	Weight (kg), mean (SD)			62.4 (9.9)
	BMI (kg/m^2^), mean (SD)			22.5 (3.6)
	**Race, n (%)**			
		White	43 (75)	
		Asian	3 (5)	
		Others	11 (19)	
	**Time since stopping hormonal contraception (month), n (%)**			
		≥3	4 (7)	
		4-6	4 (7)	
		7-9	2 (4)	
		10-12	16 (28)	
		>12	17 (30)	
		No answer	14 (25)	
	**Number of cycles per woman, mean (SD)**			3.5 (1.0)
		Women with 6 cycles, n (%)	1 (2)	
		Women with 5 cycles, n (%)	9 (16)	
		Women with 4 cycles, n (%)	17 (30)	
		Women with 3 cycles, n (%)	23 (40)	
		Women with 2 cycles, n (%)	5 (9)	
		Women with 1 cycle, n (%)	2 (4)	
**Cycle level (n=193)**				
	Cycle length (days), mean (SD)			29.5 (4.5)
	Cycle length (days), min-max			21-60
	**Anovulatory cycles, n (%)**			23 (11.9)
		Cycle length (days), min-max	21-57	
	**Ovulatory cycles, n (%)**			170 (88.1)
		Cycle length (days), min-max	24-60	
		Luteal length (days), mean (SD)	12.2 (1.9)	
		Luteal length, min-max	3-20	

### Temperature Shifts and Diagnostic Accuracy

For ovulatory cycles, the percentage of cycles with at least one temperature shift was significantly higher on the wrist skin temperature curves than that on the BBT curves (106/170, 62.4% vs 39/170, 22.9%; *P*<.001); however, the temperature shift occurred almost 2 days earlier on the BBT curves than on the wrist skin temperature curves (*P*<.001; Table 2). For anovulatory cycles, the percentage of cycles with at least one temperature shift was also significantly higher on the wrist skin temperature than on the BBT curves (17/23, 74% vs 7/23, 30%; *P*=.004).

Using the LH test as the reference standard for ovulation, the wrist skin temperature was more sensitive than the BBT (sensitivity 0.62 vs 0.23; *P*<.001) with a higher true-positive rate (106/193, 54.9% vs 39/193, 20.2%); however, it also had a higher false-positive rate (17/193, 8.8% vs 7/193, 3.6%), which resulted in a lower specificity (0.26 vs 0.70; *P*=.002; Table 2). The positive predictive value was slightly higher for the wrist skin temperature. For a temperature shift detected on a wrist skin temperature curve, there was an 86.2% probability of ovulation. On the BBT curve, this probability was 84.8%. The negative predictive value was low for both temperatures (*P*=.39). If no temperature shift was shown on a wrist skin temperature curve, there was only an 8.6% probability that this had been an anovulatory cycle. This probability on a BBT curve was slightly higher (10.9%), but the difference was not statistically significant (*P*=.74).

**Table 2:** Temperature shifts and diagnostic accuracy for wrist skin temperature and basal body temperature.

Variables				Wrist skin temperature		Basal body temperature
**Ovulatory cycles**						
	Total number of temperature shifts detected, n		240		47	
	**Cycles with ≥1 temperature shift, n (%)**		106 (62.4)^a^		39 (22.9)^a^	
		With 1 temperature shift	40 (37.7)^b^		31 (79)^c^	
		With 2 temperature shifts	31 (29.2)^b^		8 (21)^c^	
		With 3 temperature shifts	15 (14.2)^b^		0 (0)	
		With >3 temperature shifts	20 (18.9)^b^		0 (0)	
	The first day of temperature shift relative to ovulation day, mean (SD)		4.4 (2.75)		2.69 (1.89)	
**Anovulatory cycles**						
	Total number of temperature shifts detected, n		39		9	
	**Cycles with ≥1 temperature shift, n (%)**		17 (74)^d^		7 (30)^d^	
		With 1 temperature shift	5 (29)^e^		5 (71)^f^	
		With 2 temperature shifts	4 (24)^e^		2 (29)^f^	
		With 3 temperature shifts	6 (35)^e^		0 (0)	
		With 4 temperature shifts	2 (12)^e^		0 (0)	
	**Diagnostic accuracy (urine luteinizing hormone tests as standard reference;** **N=193** **)**					
		True positives, n (%)	106 (54.9)		39 (20.2)	
		True negatives, n (%)	6 (3.1)		16 (8.3)	
		False positives, n (%)	17 (8.8)		7 (3.6)	
		False negatives, n (%)	64 (33.2)		131 (67.9)	
		Sensitivity (95% CI)	0.62 (0.55-0.70)		0.23 (0.17-0.30)^g^	
		Specificity (95% CI)	0.26 (0.10-0.48)		0.70 (0.47-0.87)^g^	
		Negative predictive value (95% CI)	0.09 (0.03-0.18)		0.11 (0.06-0.17)^g^	
		Positive predictive value (95% CI)	0.86 (0.79-0.92)		0.85 (0.71-0.94)^g^	

^a^N=170.

^b^N=106.

^c^N=39.

^d^N=23.

^e^N=17.

^f^N=7.

^g^*P* values comparing wrist skin temperature and basal body temperature: *P*<.001 for sensitivity; *P*=.002 for specificity; *P=*.39 for negative predictive value; and *P*=.74 for positive predictive value.

### Correlation of the Two Temperatures

Both temperatures differed between the follicular and luteal phases, with the latter having higher temperatures (Table 3). Throughout the menstrual cycle, the wrist skin temperature was generally lower than BBT. The mean between-phase temperature change was 11% higher for the wrist skin temperature than for the BBT.

**Table 3:** Mean follicular and luteal phase temperatures for wrist skin temperature and basal body temperature.

Phases	Wrist skin temperature (°C), mean (SD)	Basal body temperature (°C), mean (SD)
Follicular phase	35.78 (0.34)	36.25 (0.16)
Luteal phase	36.07 (0.35)	36.51 (0.16)
Between-phase temperature change	0.29 (0.21)	0.26 (0.1)

**Figure 2:**
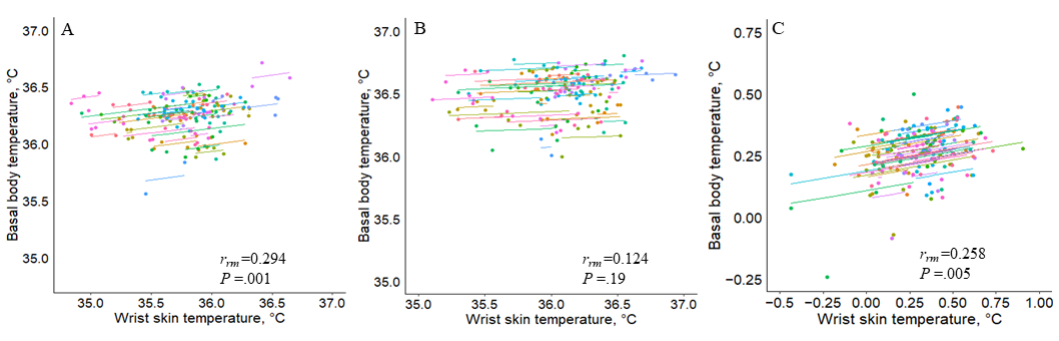
Repeated measures correlation (rmcorr) plots of mean wrist skin temperature and basal body temperature. A: correlation in the follicular phase; B: correlation in the luteal phase; C: correlation of between-phase temperature changes between the two temperatures.

Figure 2 shows the repeated measures correlation plot for the means of the two temperatures. Each dot represents the mean of the two temperatures in one menstrual cycle of a woman. Observations from the same woman are given the same color, with corresponding lines showing the repeated measures correlation fit for each woman. Positive correlations were found only in the follicular phase (*r_rm_*=0.294; 95% CI 0.117-0.454; *P*=.001). This correlation showed a minimal interindividual variation, which was reflected by the mostly parallel lines. In the luteal phase, no correlation was found between the two temperatures (*r_rm_*=0.124; 95% CI −0.061 to 0.301; *P*=.19). We found positive correlations for between-phase temperature changes measured at the two temperatures (*r_rm_*=0.258; 95% CI 0.078-0.422; *P*=.005).

### Agreement of Temperature Curves

**Figure 3:**
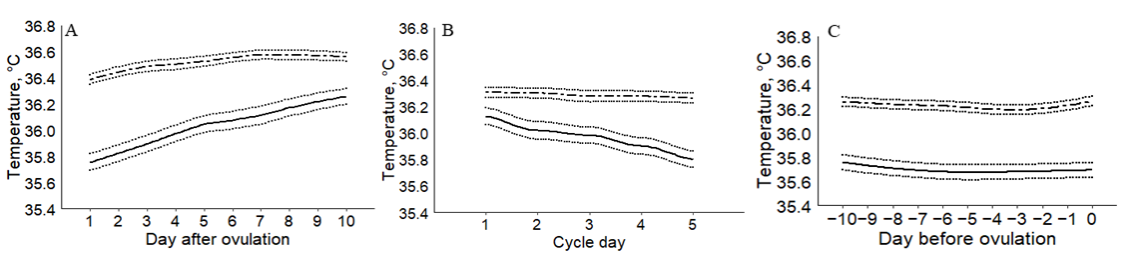
Smoothed temperature curves according to phases. A: postovulatory phase; B: menstrual phase; C: preovulatory phase. Solid and dotted lines represent wrist skin temperature and 95% CIs, respectively; dashed and dotted lines represent basal body temperature and 95% CIs, respectively.

The agreement of temperature curves was analyzed for ovulatory cycles. Figure 3 shows the smoothed curves of wrist skin temperatures and BBT during the postovulatory, menstrual, and preovulatory phases. There was no overlap between the two curves, and the agreement differed by phase. Changes in the two temperatures were not observed during the postovulatory and menstrual phases. Both temperature values increased during the postovulatory phase with a greater and more continuous increase in the wrist skin temperature (range of increase 0.50 °C vs 0.20 °C). The estimated daily difference between the two temperatures was the greatest on day 2 (0.64 °C) and the smallest on day 10 after ovulation (0.32 °C), with a mean of 0.49 °C (*P*<.001). During the menstrual phase, the wrist skin temperature exhibited a more substantial decrease (from 36.13 °C to 35.80 °C, range of decrease: 0.33 °C) than BBT (from 36.31 °C to 36.27 °C; range of decrease: 0.04 °C). The estimated daily difference between the two temperatures ranged from 0.18 °C on day 1 to 0.46 °C on day 5, with a mean difference of 0.32 °C (*P*<.001). During the preovulatory phase, there were minimal changes in both the wrist skin temperature (range: 0.09 °C) and BBT (range: 0.07 °C) and a small variation in the estimated daily difference between the two temperature values, indicating an agreement between the two curves. The mean daily difference between the two temperatures was 0.53 °C (*P*<.001).

**Figure 4:**
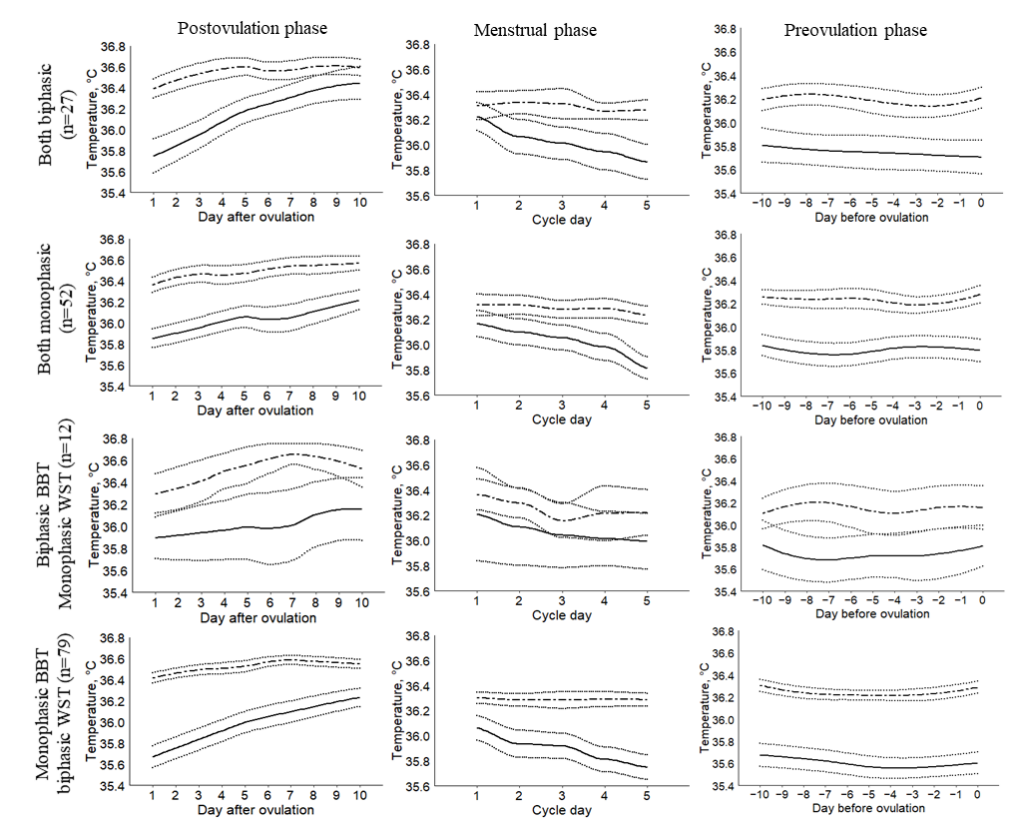
Smoothed temperature curves according to phases and patterns. Solid and dotted lines represent wrist skin temperature and 95% CIs, respectively; dashed and dotted lines represent basal body temperature and 95% CIs, respectively. BBT: basal body temperature; WST: wrist skin temperature.

Figure 4 shows the agreement based on the curve patterns. A significant overlap of the two curves was found when the BBT curve was biphasic and the wrist skin temperature curve was monophasic. However, the wide CIs could be the result of the small number of cycles in this category (n=12). The agreement between the two curves, particularly during the postovulatory phase, was highest when both curves were monophasic (n=52), with estimated daily differences between the two temperatures ranging from 0.34 °C to 0.57 °C. The most prominent disagreement during the postovulatory phase was observed when the wrist skin temperature curve was biphasic and the BBT curve monophasic (n=79), with daily differences between the two temperatures ranging from 0.32 °C to 0.74 °C. Even when both curves were biphasic (n=27), the disagreement could still be observed during the early postovulatory phase, with the largest difference between the two temperatures on day 2 after ovulation (0.67 °C). The two curves overlapped during the late postovulatory phase.

## Discussion

### Principal Findings

Continuously measured wrist skin temperature had a higher sensitivity and lower specificity for detecting ovulation than BBT measured orally. The two temperatures were significantly correlated in the follicular phase but not in the luteal phase, indicating that changes in the 2 temperatures were not coupled in the luteal phase. The wrist skin temperature curve showed a greater increase during the postovulatory phase and a greater decrease in the menstrual phase than the BBT curve. The disagreement between the temperature curves was most prominent when the BBT curve was monophasic and the wrist skin temperature curve was biphasic. Our results suggest that the continuously measured wrist skin temperature is more sensitive than BBT to detect ovulation and determine the fertile window.

This study is the first to compare the wrist skin temperature and BBT in detecting ovulation. As skin tissues are not close to major blood vessels but exposed to the environment and heat transfer from the core to surface tissue is not instantaneous, the skin temperature is generally lower than BBT but not by a fixed amount [17]. Previous studies have explored how continuously measured temperatures at various body sites change across different menstrual phases. The study by Maijala et al [10] showed that both finger skin temperature measured nocturnally and oral temperature measured upon waking up differed between the luteal and follicular phases. The average between-phase temperature change was 0.07°C higher for the finger skin temperature, and this difference was statistically significant [10]. The intestinal core temperature showed changes in the circadian rhythm over the menstrual cycle, with a higher daily mean temperature during the luteal phase than during the follicular phase, and the daily minimum temperature was at its lowest value before the LH peak [14]. Regidor et al [11] reported an accuracy of 88.8% in predicting a window of 3 days before ovulation, the day of ovulation, and 3 days after ovulation with a device that continuously measured the vaginal temperature throughout the menstrual cycle. Details about the device or the methods used to evaluate the temperature curves were not provided in their paper. Findings from our previous research confirmed that wrist skin temperature could detect menstrual phase–related temperature changes, and these changes were robust to common confounding factors known to affect BBT such as sexual activity, food intake, and alcohol consumption [9,12].

One particularly encouraging finding of this study is the higher sensitivity of the continuous wrist skin temperature than oral BBT, which indicates that continuous rather than point measurement may be more useful for pregnancy planning. Moreover, the nightly data of wrist skin temperature were handled in a more sophisticated mathematical way, allowing it to be more sensitive in detecting temperature shift in a menstrual cycle. Its lower specificity may reflect a similar trade-off between the sensitivity and specificity of any diagnostic test. One compelling argument is that specificity is more relevant for avoiding an unplanned pregnancy, as the test should have both a high true negative rate and a high negative predictive value. Although we can conclude that wrist skin temperature is more sensitive than BBT for maximizing the chances of pregnancy, neither temperature should be used as a standalone method to avoid an unplanned pregnancy given their low negative predictive values.

The difference in diagnostic accuracy between the two temperatures can be explained by the different thermal changes revealed by the two temperature curves. In particular, compared with BBT, the wrist skin temperature exhibited a greater magnitude of increase during the postovulatory phase. This allows the wrist skin temperature to be more sensitive in detecting the temperature shift. In addition, the wrist skin temperature increased in a steeper and more continuous manner. This explains why it detected more temperature shifts than BBT. Few studies have compared the two temperature curves [4,13]. Krauchi et al [4] reported a similar pattern of temperature changes in a menstrual cycle between BBT and skin temperature. In contrast, Wark et al [13] reported poor agreement between BBT and the mean upper arm skin temperature measured at 10-, 30-, 60-, 90-, and 120-minute intervals before waking.

The different modes of measurement and the different circadian rhythms of wrist skin and oral temperature, which are more pronounced during sleep [18-20], might explain the disagreement between the two temperatures. The oral temperature decreases continuously during sleep, with the lowest value occurring at approximately 5 AM, and then rises sharply after waking up [21]. Point measurements such as BBT are susceptible to variations in waking times and compliance because a single measurement is located on the sliding scale of the circadian rhythm. During the preovulatory period, the amplitude of the circadian rhythm reaches the highest value [14], which might further limit the ability of BBT to identify a temperature shift. In contrast, the circadian rhythm of wrist skin temperature features a sharp increase before lights off, a plateau at a higher temperature during sleep, and then a sharp drop immediately after rising [18,20]. Sleep propensity is accompanied by an increased skin blood flow and less cold-induced vasoconstriction, particularly in the distal skin areas that are most strongly involved in the regulation of heat loss because of their richness in arteriovenous anastomoses, thus increasing the skin temperature [22,23]. Once awake, cold-induced vasoconstriction is restored, thereby decreasing the skin temperature. In this study, participants wore a wearable device that continuously measured the wrist skin temperature during sleep. As the first 90 and the last 30 minutes of recorded data were excluded, the nocturnal steady state when the temperature was maintained at a high level was captured. As a result, the effect of the circadian rhythm was, to a certain extent, removed and the temperature changes reflected mostly the menstrual rhythm. Consequently, this measurement is more sensitive for detecting temperature shifts during menstrual cycles.

### Limitations

Our study has several limitations. First, factors that could potentially influence temperature were not evaluated. These factors include sexual activity, exercise, food intake, alcohol consumption, sleep duration and quality, and wake-up time [6]. An examination of the potential influence of these factors on the curve patterns and their agreement would have been particularly interesting. Second, because of the relatively small number of participants, we were unable to conduct subgroup analyses on the influence of BMI or cycle length on the difference in diagnostic accuracy between the two temperatures. Third, 27.4% (73/266) of the collected cycles were excluded from the final analysis because of missing measurements. The percentage of cycles with ≥30% missing wrist skin temperature was comparable with that reported in a previous study [12]. It remains unclear whether compliance would improve under real-world conditions where users are actively track their menstrual cycles. Furthermore, the participants in our study were recruited by nonrandom sampling and consisted of mostly young White women. Novel digital technology might be particularly appealing to these participants. Whether our results are generalizable to other races and real-world conditions requires further study.

### Conclusions

For women interested in maximizing the chances of pregnancy, the wrist skin temperature continuously measured during sleep is more sensitive than BBT to detect ovulation. The difference in the diagnostic accuracy of these two methods was likely attributed to the greater temperature increase in the postovulatory phase and a greater decrease during the menstrual phase for the wrist skin temperature. However, when used as a standalone method, neither of the temperatures could reliably avoid unplanned pregnancy, given the low negative predictive values. Our results underpin the importance of validation studies, especially against a standard reference test, while developing wearable devices that measure physiological parameters for women or clinicians to track menstrual cycles.

## Abbreviations

BBT: basal body temperature
LH: luteinizing hormone
